# Pulmonary Carcinoids: Diagnostic and Therapeutic Approach

**DOI:** 10.3390/cancers17172748

**Published:** 2025-08-23

**Authors:** Francesco Petrella, Andrea Cara, Enrico Mario Cassina, Lidia Libretti, Emanuele Pirondini, Federico Raveglia, Maria Chiara Sibilia, Antonio Tuoro, Stefania Rizzo

**Affiliations:** 1 Department of Thoracic Surgery, Fondazione IRCCS San Gerardo dei Tintori, 20900 Monza, Italy; andrea.cara@irccs-sangerardo.it (A.C.); enricomario.cassina@irccs-sangerardo.it (E.M.C.); lidia.libretti@irccs-sangerardo.it (L.L.); emanuele.pirondini@irccs-sangerardo.it (E.P.); federico.raveglia@irccs-sangerardo.it (F.R.); maria.sibilia@unimi.it (M.C.S.); antonio.tuoro@irccs-sangerardo.it (A.T.); 2 Clinic of Radiology EOC, Istituto Imaging della Svizzera Italiana (IIMSI), Via Tesserete 46, 6900 Lugano, Switzerland; stefania.rizzo@eoc.ch; 3 Faculty of Biomedical Sciences, Università della Svizzera italiana (USI), Via G.Buffi 13, 6900 Lugano, Switzerland

**Keywords:** carcinoid, lung, neuroendocrine, typical carcinoid, atypical carcinoid, lung resection

## Abstract

Pulmonary carcinoids (PCs) are rare tumors, with an incidence ranging from 0.2 to 2 cases per 100,000 population per year. They account for 1–2% of all invasive pulmonary malignancies and represent approximately one-fourth to one-third of all well-differentiated neuroendocrine tumors (NETs) in the body. PCs are generally classified as low- to intermediate-grade malignant tumors, further subdivided into typical carcinoid (TC) and atypical carcinoid (AC), respectively. These tumors exhibit neuroendocrine morphology and differentiation, originating from mature cells of the pulmonary diffuse neuroendocrine system. Traditionally, they are categorized as central or peripheral based on their location relative to the bronchial tree; however, they can arise anywhere within the lung parenchyma. Over 40% of cases may be detected incidentally on a standard chest X-ray, although contrast-enhanced computed tomography (CT) remains the diagnostic gold standard. Surgical resection is the treatment of choice for PCs, with the goal of complete tumor removal while preserving as much healthy lung tissue as possible. In contrast, advanced cases are typically not amenable to surgery, and medical management is focused on controlling hormone-related symptoms and limiting tumor progression. This review aims to provide an overview of the current diagnostic and therapeutic approaches to pulmonary carcinoids.

## Simple Summary

Pulmonary carcinoid tumors are a rare type of lung cancer, making up about 1–2% of all lung cancers. However, they account for about a quarter to a third of all slow-growing neuroendocrine tumors. While these tumors are uncommon in adults, they are actually the most common form of lung cancer found in children and teenagers, with the typical carcinoid being the most frequent type. The main treatment for pulmonary carcinoids is surgery, with the goal of removing the entire tumor while keeping as much healthy lung as possible. In cases where the cancer has spread too far for surgery to work, the medical community usually focuses on easing symptoms caused by hormone imbalances and slowing the growth of the tumor.

## 1. Introduction

Pulmonary carcinoids (PCs) account for 1–2% of all malignant pulmonary tumors and represent approximately one-fourth to one-third of all well-differentiated neuroendocrine tumors (NETs). PCs are classified as rare tumors, with an age-adjusted incidence ranging from 0.2 to 2 per 100,000 population per year [1]. They show a slight predominance in women over men and are more common in White individuals than in Black or other ethnic groups. PCs typically occur between the fourth and sixth decades of life, with a mean age at diagnosis of 45 years for typical carcinoids (TCs) and 55 years for atypical carcinoids (ACs). Notably, PCs are the most frequent primary lung cancer diagnosed in children and late adolescents, with TC being the predominant subtype [2]. They are generally found in never-smokers or current light smokers, although ACs are more frequently diagnosed in current or former smokers than TCs. PCs usually present as isolated lesions, but up to 5% of patients with multiple endocrine neoplasia type 1 (MEN1) may develop multiple PCs, which require differentiation from multiple pulmonary hamartomas [3,4]. Atypical carcinoids are less common than typical carcinoids, with a TC-to-AC ratio of approximately 8–10:1 [1].

PCs commonly manifest with respiratory symptoms, including persistent cough, hemoptysis, chest discomfort, wheezing, dyspnea, and recurrent respiratory infections. Contrast-enhanced computed tomography (CT) remains the imaging modality of choice for diagnosis. Compared with conventional imaging, nuclear medicine techniques provide greater specificity for the detection of typical carcinoids (TC) and atypical carcinoids (AC), allow for complete whole-body staging, and may help predict the likelihood of response to peptide receptor radionuclide therapy (PRRT). For centrally located lesions, bronchoscopy is recommended to obtain histologic confirmation; flexible bronchoscopy is generally preferred, although rigid bronchoscopy may be indicated in selected cases. Surgical resection remains the cornerstone of treatment, aiming for complete tumor removal while maximizing preservation of healthy lung parenchyma. In selected cases of purely endobronchial PCs, laser bronchoscopy can serve as a curative option, offering the benefits of rapid execution, immediate effect, and repeatability. Advanced ACs demonstrate greater biological aggressiveness than TCs and typically require a multidisciplinary therapeutic strategy, with primary goals focused on controlling symptoms—particularly those related to hormone secretion—and limiting disease progression.

While the diagnostic workup and standard treatment strategies for localized pulmonary carcinoids (PCs) are relatively well defined, significant gaps remain in the evidence base for managing locally advanced or metastatic disease. In particular, the therapeutic potential of epidermal growth factor receptor (EGFR) inhibitors such as erlotinib, antiangiogenic agents, mammalian target of rapamycin (mTOR) inhibitors, systemic chemotherapy, and peptide receptor radionuclide therapy (PRRT) has yet to be fully clarified. Addressing these uncertainties constitutes one of the anticipated contributions of the present review. In addition, this review will first examine the clinical, biochemical, and imaging-based diagnostic approach to PCs. We will then discuss therapeutic strategies for localized disease, with particular emphasis on surgical and locoregional interventions, before addressing available treatment options for locally advanced or metastatic disease.

## 2. Methods

This review outlines key aspects of the diagnostic and therapeutic management of PCs. A comprehensive literature search was conducted using PubMed, MEDLINE, and Google Scholar to identify relevant publications. The search strategy employed the following MeSH terms: “neuroendocrine lung tumours”, “typical carcinoid”, “atypical carcinoid”, “large-cell neuroendocrine lung cancer”, and “small-cell lung cancer”. Eligible studies included those reporting patient demographics, clinical presentation, and management strategies for lung neuroendocrine tumors. We considered clinical trials, cohort studies, and case–control studies published in English (2001–2025). Reference lists of selected articles were also manually reviewed to identify additional relevant studies. Exclusion criteria were opinion pieces, letters to the editor, abstracts, and preprints that had not undergone peer review.

## 3. Pathology

Pulmonary carcinoids (PCs) are tumors exhibiting neuroendocrine morphology and differentiation. They originate from mature cells within the diffuse neuroendocrine system of the lungs and are classified as low-grade (typical carcinoids, TCs) or intermediate-grade (atypical carcinoids, ACs) malignancies. Unlike more aggressive neuroendocrine tumors—such as small cell lung cancer (SCLC) and large cell neuroendocrine carcinoma (LCNEC)—PCs do not share a common genetic background or direct causal link [5]. Typical carcinoids are characterized by fewer than 2 mitoses per 2 mm^2^ and the absence of necrosis, whereas atypical carcinoids display 2–10 mitoses per 2 mm^2^ and/or areas of focal necrosis [1]. Distinguishing between these subtypes in small biopsy or cytology specimens is notably challenging and demands careful evaluation of morphological and immunohistochemical characteristics. While TCs and ACs are not reliably distinguishable in limited samples, the presence of mitotic figures or necrosis may suggest an AC diagnosis. The Ki-67 proliferation index can help exclude high-grade tumors like SCLC or LCNEC when showing low proliferative activity, but it lacks diagnostic utility for differentiating between TCs and ACs in standard biopsies [6].

PCs generally display an organoid growth pattern, often with variable sub-patterns such as nests, ribbons, trabeculae, pseudo-rosettes, papillary or follicular arrangements, paraganglioma-like structures, and pseudo-glandular formations. Tumor cells are typically polygonal or spindle-shaped, with pale cytoplasm, open chromatin, and inconspicuous nucleoli. The 2022 WHO classification of neuroendocrine neoplasms recognizes a novel subgroup of thoracic carcinoids characterized by elevated mitotic counts, a high Ki-67 proliferation index, or both. These neoplasms share certain features with atypical carcinoids but demonstrate a markedly higher mitotic activity (>10 mitoses per 2 mm^2^), indicating a more aggressive phenotype analogous to grade III well-differentiated neuroendocrine tumors of the gastrointestinal tract. The optimal pathological classification of these higher-grade carcinoids, as well as the prognostic and diagnostic value of Ki-67, remains a subject of active investigation [6].

## 4. Diagnosis

Pulmonary carcinoids often present with respiratory symptoms such as persistent cough, hemoptysis, chest pain, wheezing, dyspnea, and recurrent pulmonary infections. These symptoms are typically associated with centrally located tumors, whereas peripheral lesions are frequently asymptomatic. In rare instances, hormonal imbalances may lead to the diagnosis of a neuroendocrine tumor.

### 4.1. Biochemistry

Initial laboratory workup should include assessment of renal function, and calcium and glucose levels, as well as chromogranin A. If a functional syndrome is suspected, a 24 h urinary measurement of 5-hydroxyindoleacetic acid (5-HIAA) is advised. In patients presenting with functional hormonal syndromes, assessment should include measurement of plasma serotonin and plasma 5-hydroxyindoleacetic acid (5-HIAA). Moreover, well-differentiated pulmonary carcinoids may secrete adrenocorticotropic hormone (ACTH), potentially resulting in Cushing’s syndrome. Therefore, ACTH levels should be promptly evaluated when clinical features suggest this diagnosis.

Carcinoid syndrome occurs in approximately 2–5% of pulmonary carcinoids, generally in the presence of hepatic metastases. Cushing’s syndrome has been documented in 1–6% of cases, and when suspected, testing should include serum cortisol, 24 h urinary free cortisol, and adrenocorticotropic hormone (ACTH) levels. Notably, pulmonary carcinoids account for up to 40% of cases presenting with ectopic ACTH production. Conversely, syndrome of inappropriate antidiuretic hormone secretion (SIADH) is rare in pulmonary carcinoids but is more commonly seen in SCLC, where it occurs in about 5% of patients [7].

### 4.2. Radiology

The diagnostic imaging gold standard for pulmonary carcinoids (PCs) is contrast-enhanced computed tomography (CT). Typically, a standard lung CT protocol is employed. However, the radiologic features of PCs can be nonspecific and may resemble those of other primary lung malignancies, such as adenocarcinoma or squamous cell carcinoma. They usually appear as round or ovoid central or peripheral nodules with smooth or lobulated borders [8,9], being the central location more frequent than the peripheral [10]. PCs are often highly vascular, demonstrating high homogeneous contrast-enhancement after iodinated contrast medium administration. Calcifications are rare, and when present, they make it more difficult a differential diagnosis with hamartomas. Compared to other lung cancers, they usually exhibit a slow growth rate. In centrally located tumors, indirect signs of bronchial obstruction may be observed on CT imaging, including atelectasis, air trapping, obstructive pneumonitis, and, less commonly, bronchiectasis or pulmonary abscess [11]. Diffuse Idiopathic Pulmonary Neuroendocrine Cell Hyperplasia (DIPNECH) is a rare pre-invasive condition characterized by diffuse proliferation of pulmonary neuroendocrine cells. It is recognized as a potential precursor to carcinoid tumors. DIPNECH is more frequently diagnosed in non-smoking, middle-aged women and typically presents with symptoms such as chronic cough, dyspnea, and wheezing. High-resolution CT, particularly with expiratory imaging, is helpful in identifying characteristic findings such as mosaic attenuation, air trapping, and the presence of multiple small nodules, corresponding to tumorlets and carcinoid tumors [12].

Magnetic Resonance (MR) is not a primary lung imaging modality; however, the presence of lung nodules, especially when solid and rounded as PCs, can be an incidental finding on MRs performed for different reasons, including screening, staging, or follow-up of other malignancies, including prostate, ovarian, breast, and bone marrow cancers [13,14,15,16]. When present, PCs on MR are usually isointense to muscle on T1 weighted images and hyperintense on T2 weighted images. After gadolinium-based contrast medium injection, they resemble their hypervascular enhancement seen also on CT scans [9,17].

### 4.3. Nuclear Medicine

Nuclear medicine imaging offers a higher specificity than conventional imaging modalities for detecting TCs and ACs. It also enables comprehensive whole-body staging and can assist in predicting response to peptide receptor radionuclide therapy (PRRT) [18]. Whole-body somatostatin receptor scintigraphy (SRS) combined with thoracic single-photon emission computed tomography (SPECT)/CT can be valuable during the preoperative phase to assess nodal and distant metastases. Approximately 80% of primary tumors, especially TCs, can be detected using this technique [18,19]. Positron emission tomography with fludeoxyglucose (FDG-PET) typically shows higher standardized uptake values (SUVs) in ACs, correlating with increased proliferative activity. Therefore, FDG-PET may provide insight into tumor biology. A study by Pattenden et al. reported the sensitivity and specificity of 18F-FDG PET-CT in diagnosing mediastinal lymph node involvement in 207 patients with TCs and ACs as 33% and 94%, respectively—highlighting that a negative PET-CT does not exclude nodal metastasis in TCs [20]. When lymph node status, particularly N2 involvement, influences therapeutic decisions, further invasive staging—such as endobronchial ultrasound-guided fine-needle aspiration (EBUS-FNA), endoscopic ultrasound (EUS), or mediastinoscopy—may be necessary [21]. Where available, Gallium-68-labeled 1,4,7,10-tetraazacyclododecane-1,4,7,10-tetraacetic acid (DOTA)-somatostatin analog PET imaging offers greater sensitivity than SRS and allows for improved differentiation from rare tumors with distinct enzymatic profiles [22,23]. Other emerging techniques, such as C11-5-hydroxytryptophan PET and 64Cu-DOTATATE PET, show promise in lung neuroendocrine tumor imaging but remain limited to specialized centers [24].

### 4.4. Bronchoscopy

**Figure 1:**
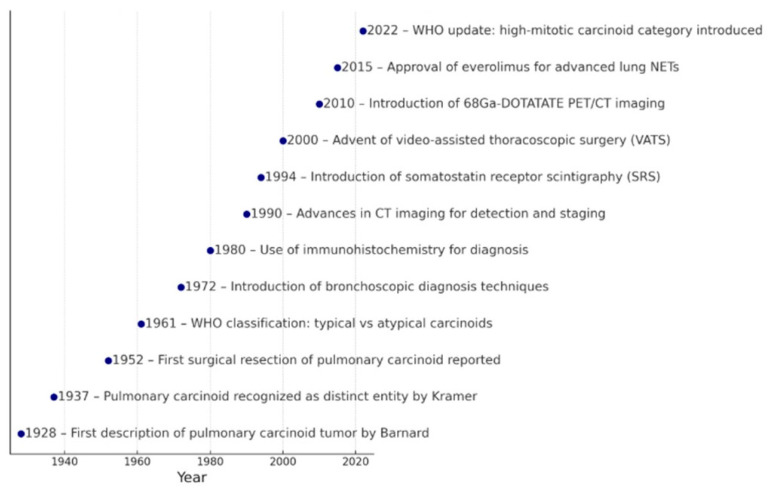
Historical progress in diagnosis and treatment of pulmonary carcinoids.

**Figure 2:**
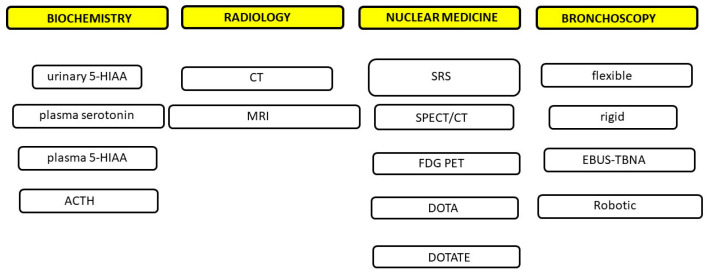
Methodologies of various diagnostic techniques. 5-HIAA: 5-hydroxyindoleacetic acid; CT: computed tomography; MRI: magnetic resonance imaging; SRS: somatostatin receptor scintigraphy; SPECT/CT: single-photon emission computed tomography; FDG PET: positron emission tomography with fludeoxyglucose; DOTA: tetraazacyclododecane tetraacetic acid; DOTATE: tetraazacyclododecane tetraacetic acid Tyr^3^-Octreotate; EBUS-TBNA: endobronchial ultrasound-transbronchial needle biopsy.

Bronchoscopy is recommended for all centrally located pulmonary carcinoids to obtain diagnostic tissue samples. Flexible bronchoscopy is generally preferred; however, in some instances, rigid bronchoscopy may be advantageous—not only for tissue acquisition but also for performing therapeutic interventions such as tumor ablation [2,25]. Central airway tumors frequently produce symptoms like dyspnea, coughing, and hemoptysis. In such cases, rigid bronchoscopy can help alleviate these symptoms while simultaneously providing a means for diagnosis. Given the indolent nature of typical carcinoids (TC), bronchoscopic tumor removal may be a suitable alternative to surgical resection in elderly individuals or those with limited functional reserve [26,27]. Biopsy of bronchial carcinoids often carries a risk of bleeding. While the safety of flexible bronchoscopy for biopsy remains a topic of debate, severe bleeding episodes have been reported. More recently, cryobiopsy has emerged as a diagnostic tool that can yield larger and better-preserved tissue samples compared to conventional forceps; however, this method may also carry a higher risk of bleeding complications [28,29]. Rigid bronchoscopy, performed under general anesthesia, allows for better control of bleeding through the use of hemostatic tools like balloons and eliminates patient coughing during the procedure, making it more comfortable. For peripheral carcinoid lesions, tissue diagnosis is usually achieved via endoscopic transbronchial biopsy or, more commonly, CT-guided transthoracic biopsy. It is important to note that small biopsy samples can complicate the differential diagnosis between typical and atypical carcinoids [26,27] [Figure 1 and Figure 2].

## 5. Treatment

### 5.1. Surgery

**Figure 3:**
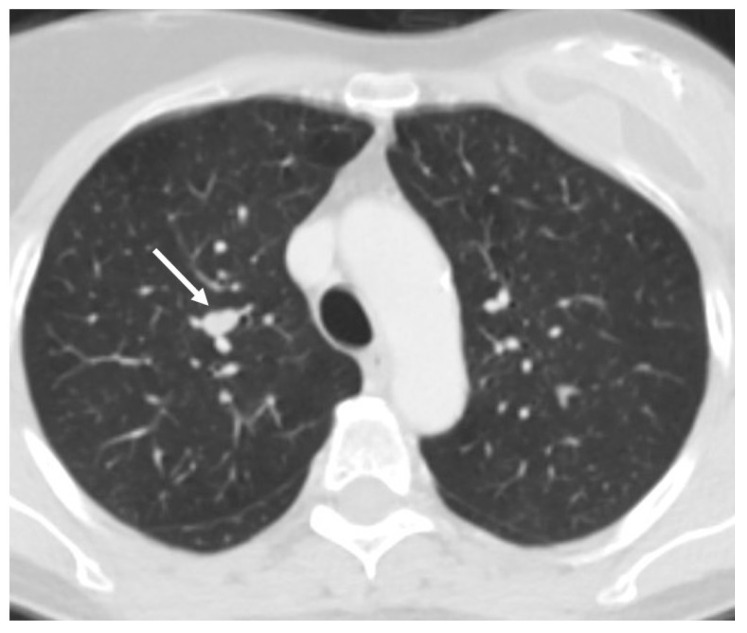
Typical carcinoid (white arrow) of the right upper lobe treated by video-assisted right upper lobectomy and lymphadenectomy.

**Figure 4:**
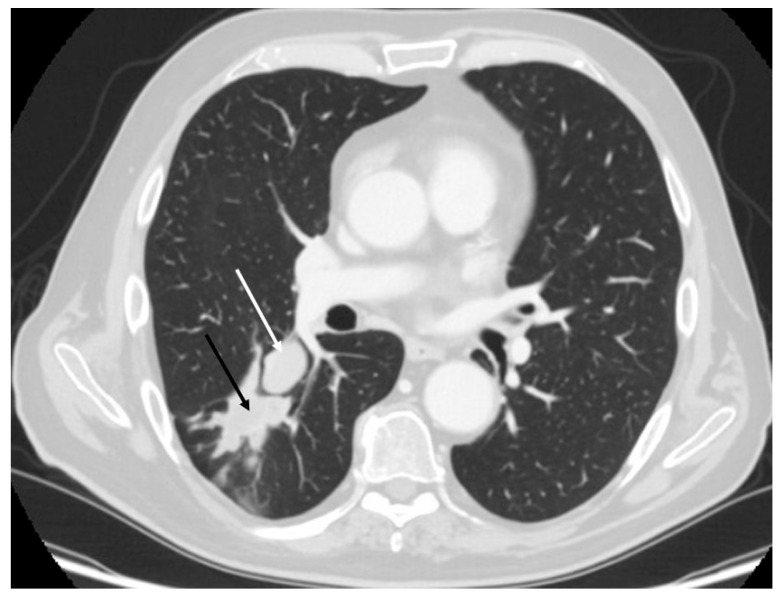
Atypical carcinoid (white arrow) of the right lower lobe complicated by post-obstructive pneumonia (black arrow), requiring right lower bilobectomy.

Surgical resection remains the primary treatment for PCs, with the goal of removing the tumor while preserving as much lung tissue as possible. The specific surgical approach depends on the tumor’s size, location, and histological type. Standard treatment typically involves complete anatomical resection along with systematic lymph node dissection. For peripheral lesions, preferred procedures include lobectomy or segmentectomy [Figure 3 and Figure 4]. Lymph node assessment should require evaluation of at least six lymph nodes or stations—three of which must be mediastinal, including the subcarinal station [1]. In patients with compromised lung function, segmentectomy is generally favored over wedge resection due to better outcomes. In cases of peripheral ACs, sublobar resections may carry a higher risk of local recurrence. For central tumors, surgical strategies should prioritize lung preservation. Bronchial sleeve resections or sleeve lobectomies are often preferred over pneumonectomy when anatomically feasible. If the tumor causes distal pneumonitis or destroyed lung, an initial endobronchial procedure to reopen the airway may be beneficial prior to reassessment for parenchyma-sparing surgery. In cases of liver metastasis, surgical resection may be performed for curative purposes, symptom relief, or tumor debulking, particularly when more than 90% of the tumor burden can be removed. Complete resection of liver metastases has been associated with 5-year survival rates exceeding 70% [30,31]. For centrally located PCs without extraluminal invasion, bronchoscopic treatment can be an effective, tissue-sparing alternative to surgery. In such cases, the pattern of tumor growth (intraluminal versus extraluminal) may be more relevant to treatment decisions than whether the tumor is classified as typical or atypical carcinoid [32,33,34]. Laser bronchoscopy may serve as a curative approach for endobronchial PCs, offering advantages such as speed, immediate effectiveness, and repeatability. Additionally, it may be combined with other treatments like radiotherapy when there is extensive intramural spread or extraluminal involvement [35,36,37].

### 5.2. Advanced Stages: Medical Management and Therapeutic Strategies

Advanced ACs are more aggressive than TCs and require a multidisciplinary approach to treatment. The main objectives are symptom control—particularly hormone-related symptoms—and slowing tumor progression. Given the variability in prognosis and the lack of curative therapies at the metastatic stage, maintaining quality of life becomes a central concern. Key considerations for medical management include the natural course of the disease without treatment, extent of metastasis, SRS uptake levels, and effectiveness of symptom control [38].

#### 5.2.1. Hormone Secretion Management

Approximately 30% of patients with advanced pulmonary carcinoids experience symptoms due to hormonal hypersecretion. Carcinoid syndrome is the most commonly associated endocrine disorder in this context. Somatostatin analogues (SSAs) are considered the first-line treatment for managing hormone-related symptoms. In rare cases (1–2%), patients may present with Cushing’s syndrome, which can be managed using medications like ketoconazole, metyrapone, etomidate, or mifepristone. If hormonal symptoms are not adequately controlled, additional therapeutic interventions—such as liver-directed procedures, combined SSA and interferon therapy, or PRRT in selected cases—should be considered. To minimize the risk of carcinoid crisis during surgical or locoregional treatments, appropriate SSA prophylaxis is recommended [39,40].

#### 5.2.2. Adjuvant Therapy Post-Surgery

There is currently no established standard for adjuvant therapy following complete surgical resection of pulmonary carcinoids. However, in patients with atypical carcinoids and lymph node involvement—especially those with a high proliferative index—postoperative adjuvant therapy may be warranted. Such decisions should be made through multidisciplinary team discussions [1].

#### 5.2.3. Tumor Control in Palliative Settings

For patients with advanced TCs or ACs with low tumor burden and proliferation rate, a conservative “watch-and-wait” strategy may be appropriate, with periodic imaging every 3–6 months. SSAs have been shown to stabilize disease in 30–70% of patients with well-differentiated neuroendocrine tumors, including pulmonary carcinoids. Long-acting formulations such as octreotide and lanreotide administered every four weeks are commonly used due to their favorable safety profiles. SSAs are recommended as first-line systemic therapy in cases with low proliferation indices and positive SRS scans. However, caution is advised in patients with aggressive disease features, and early imaging (within 2–3 months) is advised to monitor response [41]. In patients with indolent but progressive disease, various locoregional interventions targeting the primary lung tumor and metastatic sites (liver, bone, and bronchus) may be beneficial. Surgical resection of visible metastases can be considered in slowly advancing typical carcinoids or low-proliferation atypical carcinoids, particularly when local symptoms arise [42,43].

#### 5.2.4. Locoregional Therapies

Metastases to the liver, bones, or lungs can be effectively targeted using locoregional treatments such as radiofrequency ablation, with treatment success largely influenced by lesion size and anatomical location. Since liver metastases primarily receive their blood supply via the hepatic artery, targeted arterial interventions have proven effective. These include transarterial embolization (TAE) using inert particles and transarterial chemoembolization (TACE), which incorporates chemotherapeutic agents like doxorubicin. Both approaches have demonstrated radiologic response rates ranging from 33% to 73% [44]. However, current evidence does not clearly favor TACE over TAE alone in terms of clinical outcomes [45]. Additionally, novel treatments using radioactive microspheres—such as Yttrium-90—are showing promise for hepatic metastases. Combining locoregional strategies with surgery or systemic therapies may provide added benefit, especially in cases of disease progression [46,47,48].

#### 5.2.5. Peptide Receptor Radio-Targeted Therapy

Well-differentiated pulmonary carcinoids (PCs) often express somatostatin receptor subtype 2, which can be detected through imaging techniques such as Indium-111-labeled somatostatin analog scintigraphy or Gallium-68 PET scans. These imaging modalities are valuable in predicting patient response to PRRT. This therapeutic approach has shown efficacy in treating metastatic typical and atypical carcinoids, particularly using radiolabeled compounds like 90Y-DOTA-octreotide and 177Lu-DOTA-octreotide in patients exhibiting strong tracer uptake on SRS. While much of the available data is derived from single-center experiences, a large retrospective analysis of 1109 metastatic neuroendocrine tumor cases—84 of which were PCs—reported a 28% objective response rate and a 38.1% clinical improvement, with an average overall survival of 40 months. However, treatment is associated with grade 3–4 toxicities in approximately 10–33% of patients, primarily involving renal and hematologic systems. Notably, irreversible renal damage has been observed in about 9.2% of cases [49].

#### 5.2.6. Systemic Chemotherapy

Systemic chemotherapy may be appropriate for patients with advanced, unresectable, and progressive PCs. However, its overall effectiveness has been limited. The reported outcomes should be interpreted cautiously, as many studies include small, heterogeneous cohorts, outdated treatment criteria, and often lack confirmation of tumor progression at the time of enrollment. Response rates to various chemotherapeutic agents—such as 5-fluorouracil, dacarbazine, and temozolomide, whether administered alone or in combination—typically remain below 30%. Combinations including 5-fluorouracil with streptozotocin or oxaliplatin have also been explored [50,51,52]. Temozolomide, in particular, is considered a reasonable palliative option for PCs due to its relatively favorable safety profile and the extent of available data in lung NETs. It may also be useful in patients with brain metastases [53]. In one randomized study comparing 5-fluorouracil with streptozotocin versus 5-fluorouracil with doxorubicin in symptomatic carcinoids—including 22 PC cases—the overall response rate was 16%, with a median duration of response of five months. The streptozotocin-based regimen was associated with better survival outcomes, while doxorubicin offered no significant benefit [50]. A retrospective study that included 79 patients with progressive NETs—eight of whom had PCs—assessed the combination of 5-fluorouracil, streptozotocin, and cisplatin. The response rate among non-pancreatic NETs was approximately 25%, with a median time to progression of 9.1 months. Notably, one PC patient achieved partial remission, enabling surgical resection of both primary and nodal disease [54]. More recently, a randomized phase II trial involving 85 NET patients evaluated the same regimen and concluded that the addition of cisplatin did not improve outcomes [55]. Given its higher toxicity profile, cisplatin should be reserved for patients with more aggressive and advanced forms of PCs.

#### 5.2.7. mTOR Inhibitors

**Figure 5:**
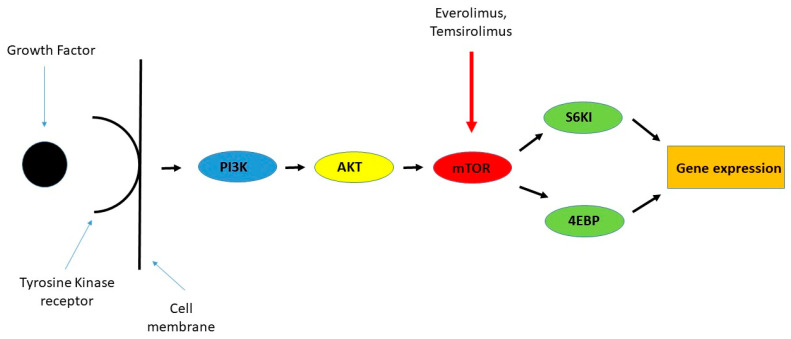
mTOR pathway.

Everolimus represents a potential therapeutic option for patients with typical (TC) and atypical carcinoids (AC) following the failure of prior treatments. The mammalian target of rapamycin (mTOR), a kinase within the PI3K signaling cascade, has been found to be activated in lung neuroendocrine tumors (NETs) [56]. Recent research has also identified PI3CA mutations in both TC and AC subtypes. The efficacy of everolimus was evaluated in the phase III RADIANT-2 trial, which included 429 patients with non-pancreatic, functioning NETs exhibiting carcinoid syndrome. Participants received either everolimus (10 mg daily) combined with long-acting octreotide (LAR) or a placebo plus octreotide LAR. The results indicated a clinically meaningful extension in median progression-free survival (PFS) by 5.1 months [57]. Further evidence was provided by the RADIANT-4 trial, a double-blind, placebo-controlled, randomized phase III study. Adult patients (aged ≥ 18) with progressive, well-differentiated, non-functional NETs of the lung or gastrointestinal tract were assigned in a 2:1 ratio to receive either everolimus (10 mg/day) or a matched placebo, alongside best supportive care. The primary endpoint was PFS, assessed via central radiology review and analyzed on an intention-to-treat basis, while overall survival served as a key secondary measure. The study found that everolimus reduced the estimated risk of disease progression or death by 52%. Although the initial interim analysis did not demonstrate a statistically significant survival benefit, it suggested a trend toward reduced mortality risk. The investigators concluded that everolimus significantly prolonged progression-free survival in patients with advanced NETs of lung and gastrointestinal origin. Additionally, the safety profile aligned with the known tolerability of the drug. Everolimus was the first targeted therapy to demonstrate consistent antitumor efficacy with manageable side effects across various NET subtypes, including pancreatic, lung, and gastrointestinal tumors [58] [Figure 5].

#### 5.2.8. Antiangiogenic Agents

The therapeutic role of antiangiogenic agents in pulmonary carcinoids PCs is still under investigation. Sunitinib, an orally administered multi-targeted tyrosine kinase inhibitor, acts on several receptors including vascular endothelial growth factor (VEGFR)-1, -2, -3, and platelet-derived growth factor receptors (PDGFR-α and PDGFR-β) [59]. In a phase II trial involving patients with carcinoid tumors, sunitinib demonstrated a modest overall response rate of 2.4%. However, a significant proportion of patients (83%) achieved disease stabilization, with a median time to progression of 10.2 months and a 1-year survival rate of 83.4% [60]. The PAZONET study evaluated the use of pazopanib, another antiangiogenic agent, as a subsequent therapy in patients with progressive metastatic NETs. It reported clinical benefit in 85% of treated individuals, which included those with pulmonary carcinoids [61]. Bevacizumab, a monoclonal antibody targeting VEGF, was assessed in a phase II study that compared it to pegylated interferon. Results showed that the bevacizumab group experienced partial tumor responses, suggesting potential activity in this setting [62].

#### 5.2.9. EGFR Inhibitors and Erlotinib

**Figure 6:**
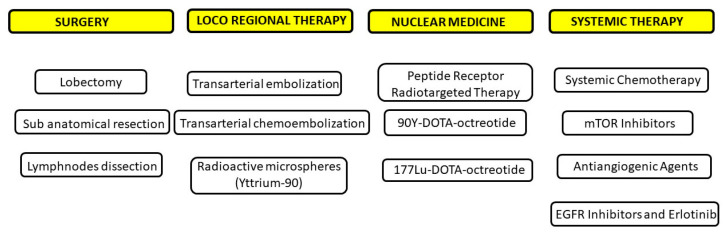
Therapeutic approach for pulmonary carcinoids.

Although the epidermal growth factor (EGF) signaling pathway is active in pulmonary carcinoid (PC) tumors, no activating mutations have been identified. Studies have shown EGFR expression in approximately 45.8% of typical carcinoids and 28.6% of atypical carcinoids. However, ErbB2 expression is absent in all cases, while ErbB3 and ErbB4 exhibit moderate to strong staining in all tumors analyzed. Genetic analysis of exons 18–21 of the EGFR gene in these tumors revealed no mutations within the tyrosine kinase domain. Nevertheless, a synonymous single nucleotide polymorphism (SNP) in exon 20 was found in 80.6% of cases. In vitro studies using lung carcinoid cell lines that express EGFR demonstrated that erlotinib, an EGFR inhibitor, can suppress tumor cell proliferation by disrupting EGFR-mediated signaling [63]. Additional pathways under investigation include the fibroblast growth factor and MET pathways, along with emerging therapeutics targeting the VEGF and PDGF signaling cascades [64] [Figure 6] [Table 1].

## 6. A Brief Overview of Other Neuroendocrine Non-Carcinoid Lung Tumors

### 6.1. Small Cell Lung Cancer (SCLC)

Small cell lung cancer (SCLC) is recognized as the most aggressive form of lung cancer, with a particularly poor prognosis. The median overall survival typically ranges from 15 to 20 months, and the two-year survival rate is estimated at approximately 5%. SCLC is distinguished by its rapid progression and early metastatic spread, along with a high rate of recurrence following initial treatment. Consequently, surgery has historically played a limited role in the management of SCLC, with the current standard of care being a combination of platinum-based chemotherapy and radiotherapy. Today, surgical resection for limited-stage SCLC is rare, accounting for only 0–6.1% of all surgically treated lung tumors. However, recent large-scale prospective cohort studies have suggested that surgery might offer benefits in carefully selected patients with early-stage disease. In contrast, locally advanced disease (stage IIIA) is generally not considered suitable for surgical intervention, as reflected in most clinical guidelines. Nevertheless, some evidence indicates that extensive lymph node dissection in N2-stage patients may positively impact survival outcomes. Despite growing interest, the precise role of surgery in limited-stage SCLC remains uncertain due to inconsistent findings across studies and the lack of recent randomized clinical trials. Given SCLC’s high propensity for metastasis and its marked sensitivity to chemotherapy, most current guidelines recommend non-surgical treatment approaches. These include platinum-based chemotherapy combined with mediastinal radiotherapy or chemotherapy alone, with prophylactic cranial irradiation reserved for more advanced cases. Surgery in early-stage SCLC may offer benefits not only in improving survival but also in securing an accurate histopathological diagnosis. This is particularly relevant when there is suspicion of mixed histology, non-small cell lung cancer (NSCLC), or rarer tumor types, allowing clinicians to adjust the therapeutic strategy accordingly and refine prognosis. More promising results for surgical intervention have been reported when surgery is integrated into a multimodal treatment regimen that includes chemotherapy and/or radiotherapy in patients with resectable disease [66]. For instance, the National Comprehensive Cancer Network (NCCN) guidelines recommend adjuvant chemotherapy even for patients staged as N0 before surgery. In cases with lymph node involvement (N+), the guidelines support either sequential or concurrent chemoradiotherapy, highlighting the more pronounced benefit of radiotherapy in pN2 disease compared to isolated N1 involvement [75].

### 6.2. Large Cell Neuroendocrine Carcinoma (LCNEC)

Large cell neuroendocrine carcinoma (LCNEC) represents a rare subtype of lung cancer, accounting for less than 1% of all cases. Approximately 40% of patients are diagnosed at a metastatic stage. Histopathological differentiation between LCNEC and small cell lung cancer (SCLC) can be challenging due to several overlapping characteristics, including the presence of necrosis, neuroendocrine morphology, positive immunohistochemical staining for neuroendocrine markers, and a high-mitotic index. Compared to other large cell non-neuroendocrine carcinomas, LCNEC generally carries a significantly poorer prognosis. Some studies have noted potential sex-based differences in overall survival, although these findings have not been consistently replicated. Similar to non-small cell lung cancer (NSCLC), the presence of mediastinal lymph node involvement in LCNEC is associated with a worse prognosis. Despite the absence of randomized controlled trials and the predominantly retrospective nature of the existing literature, there is growing recognition that LCNEC tends to respond favorably to platinum-based neoadjuvant chemotherapy. Evidence suggests that chemotherapy may also offer a survival benefit in early-stage disease, although the data remain somewhat inconsistent. Given the biological similarities and treatment responsiveness shared with SCLC, it is considered reasonable to extend platinum-based chemotherapy regimens to both advanced and early-stage LCNEC cases [75]. Current National Comprehensive Cancer Network (NCCN) guidelines recommend surgical resection for patients with non-metastatic LCNEC. Adjuvant chemotherapy, following protocols used in SCLC, is advised postoperatively. For early-stage disease (stages I to IIB), upfront surgical intervention is typically indicated. In cases of locally advanced LCNEC (stage III), a multimodal treatment approach incorporating surgery, chemotherapy, and/or radiotherapy is suggested. However, surgical management is generally not recommended for patients with stage IV disease [76].

## 7. Future Perspectives

Despite significant advances in the understanding of PCs over recent years, several areas of uncertainty remain, and multiple avenues for future research warrant exploration. The prognostic and clinical utility of Ki-67 in thoracic NET—including optimal cut-off values, reproducibility, and integration into risk stratification models—has yet to be standardized. Furthermore, the incorporation of the 2022 WHO “high-mitotic” carcinoid category into existing prognostic frameworks remains unresolved. Atypical carcinoids encompass a wide biological spectrum, yet robust biomarkers capable of reliably predicting aggressive behavior are still lacking [77].

In the field of imaging, the optimal application and sequencing of somatostatin receptor (SSTR) PET (e.g., ^68Ga-DOTATATE) versus FDG PET for prognosis, therapeutic planning, and surveillance have not been clearly defined. The potential role of liquid biopsy and circulating tumor DNA in detecting minimal residual disease, predicting recurrence, and guiding treatment decisions remains investigational [77].

From a surgical and locoregional perspective, the long-term non-inferiority of anatomic segmentectomy compared with lobectomy for peripheral typical carcinoids and selected atypical carcinoids has not been conclusively demonstrated. Similarly, the therapeutic value of systematic mediastinal lymph node dissection versus sampling in typical carcinoids, and the prognostic impact of isolated N2 disease in atypical carcinoids, require further clarification [65,66,67,68,69].

Regarding systemic therapy, the optimal first-line strategy, dosing, and treatment escalation pathways have not been specifically established for lung NET. Evidence from randomized trials defining the best regimen and sequencing remains scarce. The ideal positioning of everolimus—including timing, duration, and combination strategies—requires further evaluation, as does the real-world efficacy of antiangiogenic agents (pazopanib, sunitinib, bevacizumab). The benefit of immunotherapy in well-differentiated PCs also remains uncertain. Additionally, the prevalence and therapeutic relevance of molecular alterations (e.g., MEN1, PI3K/AKT/mTOR, RET, ALK, ROS1, HER2, KRAS) in PCs are incompletely characterized, underscoring the need for further molecular profiling to inform targeted therapy development [70,71,72,73,74].

## 8. Conclusions

PCs are neuroendocrine neoplasms characterized by distinct morphological and functional features, arising from differentiated cells of the diffuse pulmonary neuroendocrine system. These tumors are histologically classified into two categories based on their biological behavior: low-grade typical carcinoids (TCs) and intermediate-grade atypical carcinoids (ACs). Surgical excision remains the standard first-line therapy, aiming to achieve complete tumor removal while preserving optimal lung function. Atypical carcinoids, particularly in advanced stages, demonstrate more aggressive clinical behavior than typical carcinoids and often necessitate a multidisciplinary treatment strategy. Management in such cases primarily focuses on controlling hormone-mediated symptoms and mitigating disease progression. The surgical approach to limited disease-SCLC should be standard lobectomy with lymphadenectomy which provides the best overall survival, in particular when compared to lesser resection such as wedge resection (39); on the other hand, the role of pneumonectomy in SCLC is unclear and, taking into consideration the disease’s biology and the high-risk postoperative course, it should be avoided even in salvage settings.

## Figures and Tables

**Table 1:** Recent studies on pulmonary carcinoids treatments.

Title	Authors	Year	Domain/Study Design	Sample Size	Key Findings	Pub Med Reference
Prognosis of unresected versus resected early-stage pulmonary carcinoid tumors ≤ 3 cm in size: a population-based study [65]	Li et al.	2024	Surgery/Retrospective cohort study	4552 patients	Surgical resection of small PCs is associated with a survival advantage over observation. In early PCs ≤ 1 cm in diameter, observation may be considered in patients with high risk for surgical resection.	PMID: 38855831
Impact of surgical extent on survival in pulmonary typical carcinoids: a retrospective analysis [66]	Bertolaccini et al.	2025	Surgery/Retrospective cohort study	524 patients	While lobectomy remains the most commonly performed procedure for TCs, none of the evaluated surgical approaches significantly influenced OS or DSF. Notably, mitoses and nodal metastases emerged as key negative prognostic factors.	PMID: 40560517
Segmentectomy and wedge resection are equivalent for the treatment of early-stage pulmonary carcinoid tumors: a retrospective cohort study [67]	Qi et al.	2024	Surgery/Retrospective cohort study	2078 patients	There was no significant difference in survival between the sublobar resection and lobectomy groups in either the TC or AC tumor groups.	PMID: 39085450
The role of wedge resection and lymph node examination in stage IA lung carcinoid tumors [68]	Li et al.	2024	Surgery/Retrospective cohort study	2029 patients	For early-stage PCs, wedge resection was not inferior to anatomical resection in terms of OS.	PMID: 39444868
Recurrence rates and patterns after radical resection of lung carcinoids [69].	Askildsen et al.	2024	Surgery/Retrospective cohort study	217 patients	In both ACs and TCs, most recurrences were distant and occurred in patients with a resection margin less than 2 cm. AC recurs more often than TC, even in patients without nodal involvement at surgery.	PMID: 39272839
Lung carcinoid tumors with potentially actionable genomic alterations and responses to targeted therapies [70]	Waliany et al.	2025	Oncology/Retrospective cohort study	321 cases of lung carcinoids profiled by NGS	Patients with advanced lung carcinoids harboring actionable genomic alterations can derive meaningful benefit from genotype-matched targeted therapies.	PMID: 40234130
Current and emerging strategies for the management of advanced/metastatic lung neuroendocrine tumors [71]	Rutherford et al.	2024	Oncology/Review article	Not applicable	Discusses somatostatin analogs, targeted therapy, chemotherapy, and immunotherapy as treatment options.	PMID: 38281845
The first case report of effective treatment with sotorasib for metastatic atypical lung carcinoid harboring KRAS G12C mutation and aggressive disseminated lung metastasis: a case report [72]	Saiki at al.	2023	Oncology/Case report	1	The first case of KRAS G12C mutation-positive atypical carcinoid that was successfully treated with sotorasib.	PMID: 38299192
Management of typical and atypical metastatic lung carcinoids: present and future perspectives [73]	Rodrigues et al.	2025	Oncology/Review article	Not applicable	Emphasizes dual objectives of controlling tumor growth and managing symptoms; discusses systemic treatments and locoregional options.	PMID: 39110397
Treatment of advanced BP-NETS with lanreotide autogel/depot vs placebo: the phase III SPINET study [74]	Baudin et al.	2024	Oncology/Phase III, double-blind randomized controlled trial	77 patients	Showed clinical benefit of lanreotide in advanced typical carcinoids.	PMID: 38913539

Legend: OS: overall survival; DSF: disease-free survival; PCs: pulmonary carcinoids; TC: typical carcinoid; AC: atypical carcinoid.

## Data Availability

Data are available on request.

## Abbreviations

The following abbreviations are used in this manuscript:

PC	Pulmonary Carcinoid
TC	Typical Carcinoid
AC	Atypical Carcinoid
CT	Computed tomography
MEN1	Multiple endocrine neoplasia type 1
SCLC	Small cell lung cancer
LCNEC	Large cell neuroendocrine carcinoma
5-HIAA	5-hydroxyindoleacetic acid
ACTH	adrenocorticotropic hormone
SIADH	syndrome of inappropriate antidiuretic hormone secretion
DIPNECH	Diffuse Idiopathic Pulmonary Neuroendocrine Cell Hyperplasia
PRRT	peptide receptor radionuclide therapy
SRS	somatostatin receptor scintigraphy
SPECT	single-photon emission computed tomography
FDG-PET	Positron emission tomography with fludeoxyglucose
EBUS-FNA	endobronchial ultrasound-guided fine-needle aspiration
EUS	endoscopic ultrasound
DOTA	Gallium-68-labeled 1,4,7,10-tetraazacyclododecane-1,4,7,10-tetraacetic acid
TAE	transarterial embolization
TACE	transarterial chemoembolization
SSA	Somatostatin analogue
VEGF	Vascular endothelial growth factor
PDGFR	platelet-derived growth factor receptors
EGF	epidermal growth factor
SNP	single nucleotide polymorphism
